# A Mutational Hotspot and Strong Selection Contribute to the Order of Mutations Selected for during *Escherichia coli* Adaptation to the Gut

**DOI:** 10.1371/journal.pgen.1006420

**Published:** 2016-11-03

**Authors:** Marta Lourenço, Ricardo S. Ramiro, Daniela Güleresi, João Barroso-Batista, Karina B. Xavier, Isabel Gordo, Ana Sousa

**Affiliations:** Instituto Gulbenkian de Ciência, Oeiras, Portugal; University of Houston, UNITED STATES

## Abstract

The relative role of drift versus selection underlying the evolution of bacterial species within the gut microbiota remains poorly understood. The large sizes of bacterial populations in this environment suggest that even adaptive mutations with weak effects, thought to be the most frequently occurring, could substantially contribute to a rapid pace of evolutionary change in the gut. We followed the emergence of intra-species diversity in a commensal *Escherichia coli* strain that previously acquired an adaptive mutation with strong effect during one week of colonization of the mouse gut. Following this first step, which consisted of inactivating a metabolic operon, one third of the subsequent adaptive mutations were found to have a selective effect as high as the first. Nevertheless, the order of the adaptive steps was strongly affected by a mutational hotspot with an exceptionally high mutation rate of 10^−5^. The pattern of polymorphism emerging in the populations evolving within different hosts was characterized by periodic selection, which reduced diversity, but also frequency-dependent selection, actively maintaining genetic diversity. Furthermore, the continuous emergence of similar phenotypes due to distinct mutations, known as clonal interference, was pervasive. Evolutionary change within the gut is therefore highly repeatable within and across hosts, with adaptive mutations of selection coefficients as strong as 12% accumulating without strong constraints on genetic background. *In vivo* competitive assays showed that one of the second steps (*focA*) exhibited positive epistasis with the first, while another (*dcuB*) exhibited negative epistasis. The data shows that strong effect adaptive mutations continuously recur in gut commensal bacterial species.

## Author Summary

The relative contribution of random loss and migration versus *de novo* mutation to the overall diversity of the gut microbiota is far from understood. Population sizes of bacterial communities inhabiting the gut can be very large and therefore, both weak and strong effect beneficial mutations theoretically have the opportunity to contribute to adaptation. Here, by discretizing the adaptive steps that occur during colonization by a gut commensal, we uncover a mutational hotspot, large effect mutations and different forms of natural selection as the core ingredients to the emergence of diversity in this environment. We show that, on occasion, strong periodic selection can create and reduce diversity but then recurrent mutation generates new adaptive variants that compete for fixation. Selection for keeping particular adaptive variants at low frequency, balancing selection, was also shown to be pervasive. Unexpectedly, given the complexity of the gut ecosystem, we find a highly repeatable evolutionary process, motivated by a mutational hotspot and strong effect adaptive mutations continuously recurring in commensal bacterial species.

## Introduction

The composition of the gut microbiota can exert a strong influence on host physiology, behavior and health. Time series data have shown that the gut microbiota typically comprises a diverse community of species and that reduction of such diversity is frequently associated with illness [1]. Less studied, but potentially as important, is the diversity at the level of each species [2,3]. In fact, studies providing an understanding on how intraspecific variation in the microbiota emerges and changes over time are lacking [4]. Therefore, important questions such as whether the extant intra-species diversity is mainly due to migration and genetic drift or the result of natural selection on new mutations remain unanswered [5]. While some mutations segregating in natural populations may be neutral, the large size of bacterial communities inhabiting the mammalian gut suggests that here, polymorphism is more likely to result from deterministic forces [6].

The importance of natural selection and its strength *versus* other evolutionary processes in shaping intra-species variation in the guts of hosts living in their natural environments is hard to dissect. Direct measurements of selective effects of spontaneously emerging mutations in this environment are extremely rare due to its complex nature. Nevertheless, even mutations with very weak adaptive effects, thought occur most frequently [7], could substantially contribute to a rapid pace of bacterial evolutionary change in the gut. Here, using experimental evolution of *E*. *coli* colonizing a natural environment, coupled with whole genome sequencing and *in vivo* competitive assays, we unravel some of the targets of adaptive evolution and the strength of the effects of beneficial mutations *in vivo*. Mouse colonization models offer a great opportunity to observe the emergence of diversity, test its repeatability amongst different hosts and measure the strength of natural selection in bacterial populations comprising the gut microbiota. Using a common model of gut colonization, we show that all classical forms of natural selection, *i*.*e*. periodic selection, balancing selection and clonal interference are ubiquitous, and contribute to strain variation within the gut. Furthermore, we were able to i) quantify important evolutionary parameters in this system, such as the effect of second step mutations, ii) evaluate the repeatability of evolution and iii) assess possible constrains on the order of the observed adaptive events.

## Results

### Multiple targets and a high degree of parallelism characterize the second steps of *E*. *coli* gut adaptation

By following the evolution of a commensal *E*. *coli* strain inhabiting the guts of streptomycin treated mice, we previously observed the emergence and spread of adaptive mutations within only 3 days of colonization [8]. The first step of adaptation targeted a single locus and consisted of the selective inactivation of the *gat* operon, which enables *E*. *coli* to metabolize galactitol [8]. Distinct alleles conferring this phenotype, with similar selective effects (8 ±0.01% (2SE) benefit), recurrently emerged in all independently evolving *E*. *coli* populations recovered from different hosts. Using this experimental system, we have now studied the subsequent steps of adaptation. We colonized 15 mice with a clonal population of *E*. *coli* carrying the first beneficial phenotype (inability to metabolize galactitol). This phenotype is conferred by a single base pair insertion into the coding region of the *gatC* gene, which codes for a subunit of the galactitol transporter, to thus prevent galactitol uptake. The colonizing population was also made dimorphic for a fluorescent marker to enable the emergence of further adaptive changes to be determined and their effects measured by competitive fitness assays [9].

**Fig 1:**
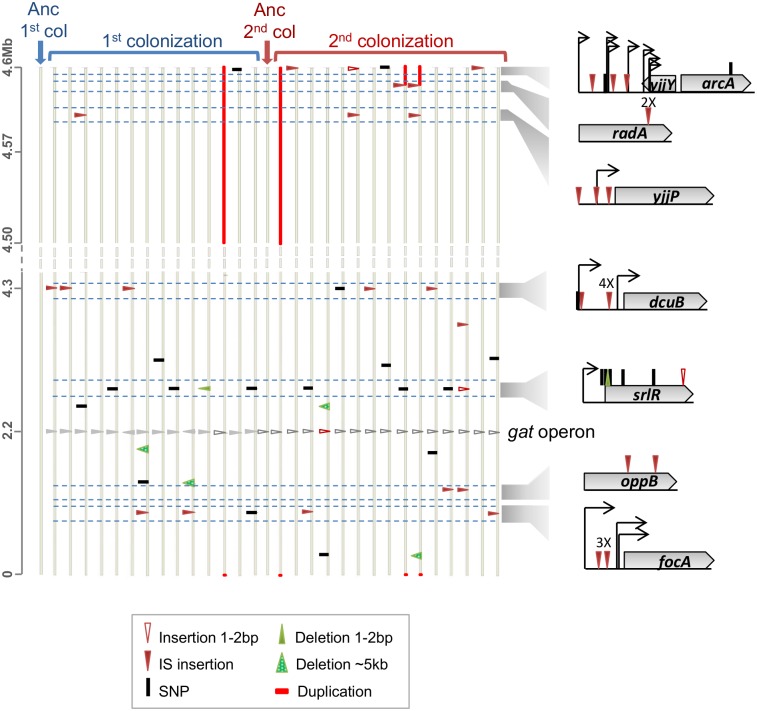
The genetic basis of the 2^nd^ step of adaptation involved 7 parallel mutational targets. Mutations were identified by whole genome sequencing of 29 evolved clones: 14 independently evolved clones from an ancestral strain (first colonization) [8] and 15 independently evolved clones from an adapted clone carrying a single beneficial mutation (second colonization). For simplicity, the genomes are represented linearly (vertical bars). The genomic context of the parallel mutations is represented on the right. Type and position of mutations are shown as triangles for insertions and deletions and small vertical bars denote single nucleotide polymorphisms (SNPs). Four duplications are depicted as red horizontal bars. See the symbol legend for other events. Regions of parallel mutation are highlighted. Numbers above marked mutations represent the number of clones where a particular mutation was detected at the same position.

To ask whether single or multiple genetic targets underlie the second step of adaptation, and to determine how repeatable evolution is, we performed whole genome sequencing (WGS) of 15 independently evolved clones (sampled at day 24 post colonization, see Fig 1 and S1 Table).

A total of 30 mutations were detected, including 12 mutations in coding regions, 13 mutations in intergenic regions, as well as 3 large duplications and 2 large deletions. From the mutations observed in coding regions, 3 were non-synonymous, 1 was a nonsense mutation, and 6 mutations involved insertion sequence (IS) insertions. Finally, 8 IS insertions and 5 small insertion deletion mutations occurred in intergenic regions. The average number of mutations per sequenced clone was 2, a number similar to that observed in clones sampled from the first colonization (2.3 [8], *n* = 14, Fig 1).

To determine the likely targets of adaptation we compared the mutations occurring in the clones sampled from both colonizations. As shown in Fig 1, some mutational targets were similar between the first and second colonizations. Pooling all the clones, 7 new targets of mutation (*srlR*, *arcA*, *yjjP*, *oppB*, *radA*-dup, *dcuB* and *focA*) were recurrently detected in different mice, revealing their adaptive nature [10]. The most frequent was *srlR*, a locus that codes for the repressor of the sorbitol operon; followed by insertions in the intergenic region upstream of *dcuB* and *focA*, which code for membrane transporters of C4-dicarboxylates (e.g. fumarate) and formate, respectively [11,12]. The regulatory region of *arcA*, a dual transcriptional regulator predominantly involved in controlling the respiratory flexibility of *E*. *coli* [13,14], was also highly targeted. Another frequent mutation involved a large duplication (ranging from 34Kb to 157Kb). Two additional targets were observed less frequently: the promoter region of *yjjP*, which codes for a membrane protein of unknown function [15], and the coding region of *oppB*, which codes for a component of the oligopeptide ABC transporter [16]. These two loci were targeted by IS element insertions.

### Transposon insertions drive adaptation to the mouse gut by fine-tuning gene expression

Half of the mutations identified in the sequenced clones (*n* = 29) were caused by IS insertions (Fig 1 and S1 Table), specifically IS5, IS1,IS2 and IS186, which have a high rate of transposition, between 10^−6^ and 10^−5^ per element per generation, in *E*. *coli* [17]. In fact, IS insertions occurred in 6 out of the 7 targets of adaptation identified through parallelism. Among these insertions, 78% were located in regulatory regions (S1 Fig), suggesting that mutations altering gene regulation are an important driver of adaptation in these populations [18]. Only one of the second step mutations involving an IS insertion likely caused a loss of function (insertion of an IS element in the coding region of *oppB*). The phenotypic effects of IS adaptive insertions in the regulatory regions of *focA*, *dcuB*, *arcA*, *yjjY* and *yjjP* were determined by comparing gene expression of representative mutants evolved during gut colonization with that of the ancestral clone (see Material and Methods). Since *E*. *coli* in the gut experiences variation in the amount of oxygen conditions [13,14], gene expression was assayed both in the presence and absence of oxygen.

**Fig 2:**
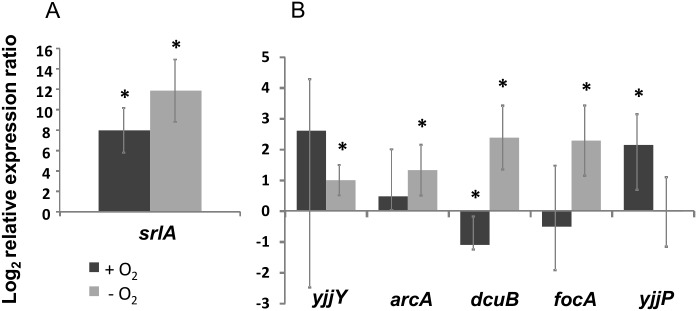
Transposable elements modulate gene expression. The graph represents the log_2_ relative expression rate of each gene in the evolved clone in relation to that of the ancestral clone. This was obtained by RT-qPCR and analyzed according to the method described in [19], using *hfq* as an housekeeping gene. The expression rate is above 0 for evolved clones where a SNP in *srlR* (A) or an IS insertion in the depicted genes (B) caused an increase in gene expression (in relation to the ancestor); if this value is below 0 gene expression was decreased (in comparison to the ancestor). Asterisks indicate significant differences at the level *P* < 0.05 (unpaired T-test). Effects of IS insertions on gene expression were conditional on oxygen availability. Error bars represent 2 times the standard error. Representative clones used for RT-qPCR measurements were the following (see S1 Table): 18YFP (*focA srlR*), 22YFP (*dcuB*), 25YFP (*yjjP/yjjQ radA insX-insA*), 29CFP (*arcA*) and the ancestral strains DM08-YFP and DM09-CFP.

In anaerobic conditions the transcription level of *arcA*, *focA* and *dcuB* was higher in the evolved clones than in the ancestor. In aerobic conditions the effect of IS insertions on expression was more variable, ranging from a 1-fold reduction of *dcuB* expression to a 2-fold increase in the expression of *yjjP* (Fig 2A).

Mutational targets provide clues on the traits under selection in a given environment. Three of the targets identified (*arcA*, *focA* and *dcuB*) are related with microaerobic/anaerobic metabolism. For example, fumarate (transported by DcuB) has been described as the most important anaerobic electron acceptor in the intestine of streptomycin-treated mice model [14], whereas formate (transported by FocA) is the signature compound of anaerobic metabolism for *E*. *coli* [20]. The gut is described as a microaerobic environment [13,14] where *E coli* can take profit of its respiratory flexibility. ArcA is known to be an important regulator of this trait and to play an important role in the ability of *E*. *coli* to colonize [13,14]. Specifically, ArcA is a global repressor of carbon oxidation pathways [21], repressing the expression of 74 operons and inducing the expression of 11 operons (including *focA*) under anaerobic conditions. Interestingly, the 4 large duplications, observed in different hosts, involved a genomic region that includes *arcA*. Since duplications can raise gene dosage we hypothesize that increased expression of this gene constitutes a major beneficial phenotype.

In sum, the mutational targets indicate that *E*. *coli* is adapting to the intestinal tract by tuning the expression of a global respiratory regulator (*arcA*) and changing its carbohydrate metabolism and transport (*srlR*, *dcuB*, *focA* and *oppB*). Consistent with this metabolic adaptation, several gut-adapted clones have an increased fitness when competing with the ancestral in media containing carbon sources known to be present in the intestine [22]. This increased competitive ability occurs both in the presence and absence of oxygen (S2 Table).

Another interesting example of gain-of-function mutations, not involving transpositions, occurred at the *srlR* locus (which codes for the transcriptional repressor of the sorbitol operon). Around half of the mutations observed in this gene (considering the 29 clones from the two colonizations) were either single base pair insertions or nonsense mutations, which thus presumably inactivated this gene. Indeed the expression of *srlA* (the first gene of the sorbitol operon) was increased 12-fold and 8-fold in the absence and in the presence of oxygen, respectively (Fig 2B), in the gut-adapted clones, supporting the hypothesis that mutations in *srlR* abrogated expression of this repressor and lead to increased expression of the sorbitol operon. Consistently, the mutants carrying the *srlR* mutation have an advantage when competing for sorbitol (S2 Table).

### The order of adaptive steps reflects both their effects and mutation rate heterogeneity

**Fig 3:**
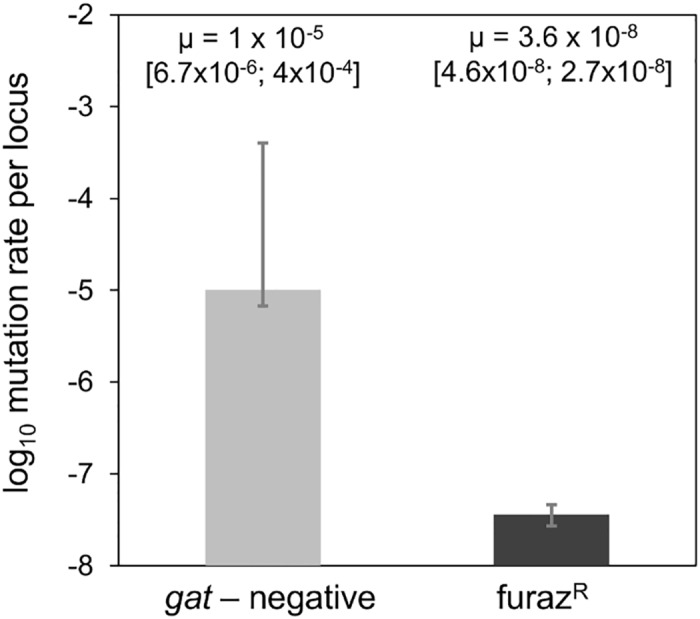
The *gat* locus is a mutational hotspot. The graph shows the log_10_ mutation rate (μ) (per locus, per generation) for each phenotype: *gat*-negative (grey bar) and furazolidone resistance (black bar) and their respective confidence intervals. Resistance to furazolidone is conferred by the inactivation of the *nfsA* gene. The values of μ were estimated by performing fluctuation assays and analyzing the results using the maximum likelihood approach implemented in FALCOR [25]. The mean frequency of spontaneous *gat-*negative mutants is ~300 times higher than the frequency of spontaneous resistants to furazolidone. After correcting for the difference between loci sizes the frequency of *gat*-negative phenotype is still ~38 times higher than the frequency of furazolidone resistance.

The order of the mutational steps along an adaptive walk is influenced by the mutation rate, the fitness effects of mutations and/or possible epistatic interactions as well as the stability of the environment [23,24]. We investigated whether these processes play a role in *E*. *coli* adaptation to the mouse gut. We measured the spontaneous mutation rate to the functional inactivation of the *gat* operon (μ), using a fluctuation assay in rich medium (LB), and found a μ of 10^−5^ (95% CI, [6.7x10^-6^, 4x10^-4^]) per locus per generation (Fig 3).

We compared this rate with the rate of inactivating mutations occurring at another locus of the genome (*nfsA*), which results in resistance to furazolidone (furaz^R^). For this locus we found a rate of 4x10^-8^ (95% CI, [4.6x10^-8^, 2.7x10^-8^]), far closer to the expected rate for loss of function mutations (10^−8^ per gene per cell division [26]). After correcting for locus size differences, the inactivation rate of the *gat* operon is one order of magnitude higher than that of *nsfA*, which is taken as a random locus. The estimation of the mutation rate in the fluctuation test performed above assumes that the mutations causing the phenotype for which μ is being determined are neutral. To account for a potential bias in μ estimate, which might have resulted from a lack of neutrality of the *gat*-negative phenotype in LB, we determined its fitness effect when in direct competition with the *gat*-positive ancestor in this environment. The *gat*-negative phenotype was found to be deleterious *in vitro*, with an estimated selection coefficient of -0.06 ±0.01 (2SE). A similar competition between a *nfsA* knock mutant and the wild type bacterium resulted in a selection coefficient of -0.007±0.005 for the mutant, thus the knock out of this gene is very close to neutrality. Our estimate of μ is therefore conservative, since the strong deleterious effect of the phenotype being scored can only cause an underestimation of μ. These results support the conclusion that the high rate of spontaneous inactivation of the *gat*-operon could have been an important contributory factor for the emergence of the *gat-*negative phenotype as the first adaptive event to occur.

**Fig 4:**
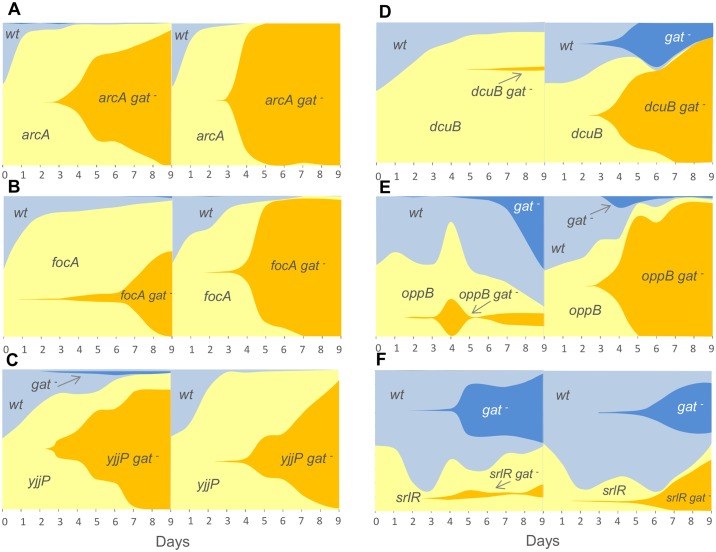
The mutations occurring during the second step of adaptation show beneficial effects in the wild type background (*gat*-positive). Graphical representation of the frequencies of each mutant (labelled with a *yfp* allele) carrying one of the second step mutations: (A) *arcA*, (B) *focA*, (C) *yjjP*, (D) *dcuB*, (E) *oppB* and (F) *srlR* and the ancestor (labelled with a *cfp* allele). Shaded areas are proportional to the frequencies of each phenotype. Increasingly darker tones of yellow or blue represent newly generated *gat*-negative phenotypic mutants in the respective fluorescent background. *gat*-negative mutants (likely originating from multiple mutations and typed for the phenotype) invade both genetic backgrounds, indicating that its beneficial effect could be observed both in the background of the first and second step mutations. In this sense no historical contingency along the adaptive walk is expected since the fixation of any of the second step mutations could not prevent the emergence and spread of the *gat*-negative phenotype.

**Fig 5:**
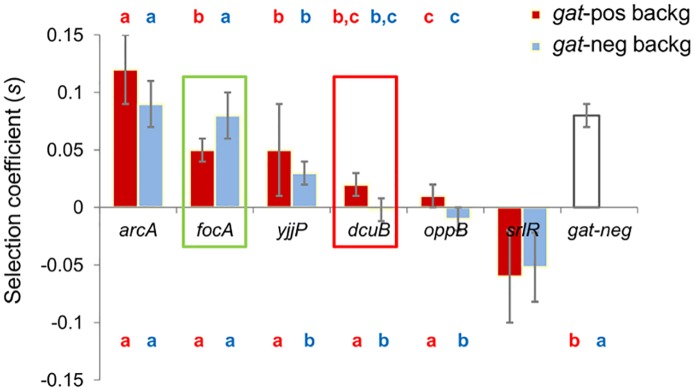
The second step mutations show variable effects both within and between backgrounds (*gat*-positive and *gat*–negative). All second step mutations except for the *srlR*, are either beneficial or neutral in the two backgrounds (red for the *gat*-positive background and blue for the *gat*-negative background). The selection coefficient of each mutation is an average of 4 independent competitions (except for *yjjP* on the *gat*-positive background where *n* = 3. See also S2 Fig) and was estimated as described in the Methods. The time interval used to estimate the selection coefficient was 3 days in all cases, except for the *arcA* mutation in the *gat*-positive background (since it reached a very high frequency after 2 days), and for the *srlR* mutation in the *gat*-negative background where 1 day was used (since it shows FDS- see Fig 6). Letters above the bar chart indicate which selective effects are significantly different from each other in the respective background (ANOVA with Tukey *post-hoc* correction for multiple testing. Please see S3 Table for the significance associated with each pairwise comparison). Letters below the bar chart indicate which selective effects are significantly different from the *gat*-negative mutation (ANOVA Contrasts, please see S3 Table for the *P* value associated with each pair wise comparison). Error bars (2SE). Rectangles (green and red) denote the mutations with a significantly different effect in the *gat*-positive versus *gat*-negative backgrounds (*i*.*e*. mutations showing an epistatic interaction with the background, green rectangle denotes positive epistasis and red negative epistasis. *P* < 0.05, Mann-Whitney-Wilcoxon Test).

Heterogeneity in the mutation rate does not exclude a possible contribution of direct or epistatic selection to the order of the adaptive events. To test for this, and to determine the selective effects of beneficial mutations in the gut, we estimated the selection coefficients of 6 out of the 7 second step mutations when each occurs either in a wild-type (*gat*-positive) or in an evolved (*gat*-negative) background. This was done through *in vivo* competitive fitness assays against the ancestors of the first (*gat*-positive) (Figs 4 and 5) and second colonizations (*gat*-negative) (Fig 5). The *radA*-dup mutation was not tested, as this duplication was highly unstable during *in vitro* manipulation. We note that despite the allelic variation within each target of adaptation, only one allele representative of each locus was tested. While for the *gat* locus we have previously shown that different alleles have equivalent selective effects [8,27], we acknowledge that for the second targets of adaptation, different alleles may have different phenotypes.

We found substantial variation for the selective effects of these mutations in both backgrounds (*P* = 6 x 10^−6^ for the *gat*-positive background and *P* = 6 x 10^−6^ for *gat*-negative background, ANOVA). This contrasts with the effect of the mutations responsible for the first step of adaptation (for which a selective effect, *s*_*gat-*neg_ = 0.08 ± 0.01, was estimated [8], Fig 5). Most mutations showed a weaker competitive advantage on the *gat*-positive background than those causing the first adaptive step (ANOVA with Tukey *post-hoc* correction for multiple testing, Fig 5 and S3 Table). Interestingly the effect of the *arcA* mutation was significantly larger than the first step mutations (*P* = 0.01, ANOVA Contrasts). These results indicate that alongside differences in the mutation rate, selection might also have contributed to the order of the adaptive steps.

Remarkably, the mutation inactivating the *srlR* locus, leading to constitutive expression of the sorbitol operon (Fig 2B), steadily decreased in frequency (*s*_*srlR*_ = -0.03) during the first days of competition (Fig 4F). It was subsequently maintained at low frequency, an observation that would not be expected for a strictly deleterious allele. This mutation also emerged in multiple, independent populations (Fig 1), all of which suggests that *srlR* is a likely target for balancing selection, a hypothesis further investigated below.

Importantly, we observed the appearance of the *gat*-negative phenotype on the ancestral background, and in all backgrounds carrying a single second step mutation (Fig 4). This strongly suggests that high repeatability and little historical contingency on genetic background, accompany the evolutionary path taken by *E*. *coli* during gut colonization. Here we refer to historical contingency as the situation where the beneficial effect of a focal mutation is contingent on the ancestral background since it becomes deleterious in an evolved background, thus limiting the number of evolutionary paths. [28].

To determine whether epistatic interactions occur between the mutations, and how the first and second steps differ in terms of magnitude, we further measured the effects of these second step mutations in the *gat*-negative background. We observed that mutations in *arcA*, *yjjP* and *oppB* had a similar effect in *gat*-negative and *gat*-positive backgrounds, whereas the fitness effect of mutations in *dcuB* and *focA* differed (*P* = 0.029 for both of the mutations. Mann-Whitney-Wilcoxon). *dcuB* showed negative epistasis, since its effect is undistinguishable from neutrality on the more adapted background (the *gat*-negative) while it is beneficial (0.02 ± 0.01) in the less adapted background (*gat*-positive). The high frequency of emergence of mutations in *dcuB* during the evolution experiment, however, strongly support its having a beneficial effect in the *gat-*negative background, suggesting that *dcuB* is, in fact, a likely target for frequency-dependent selection. Conversely, the mutation in *focA* had a significantly higher effect in the more adapted, *gat*-negative, background. This provides an interesting example of positive epistasis, a phenomenon which, though documented, occurs much less frequently than that of negative epistasis [29–31].

Consistent with theoretical models of adaptation towards a fitness optimum, several empirical studies have found that the effects of beneficial mutations tend to decrease as adaptation proceeds [32–34]. Interestingly, we have found that two of the second step mutations (*s*_*arcA*_ = 0.09 ± 0.02 and *s*_*focA*_ = 0.08 ± 0.02) were as large as the first step (*s*_*gat-*neg_ = 0.08 ± 0.01) (*P* = 0.01 and *P* = 0.05, respectively. ANOVA Contrasts). This observation suggests that the gut maybe more complex than a more strictly constant *in vitro* environment or that there are multiple discrete traits to optimize even in a constant environment. A key distinction between a fixed and a moving landscape is that in the first the adaptive walk is short and strong diminishing returns are expected. In a moving landscape [35–37], adaptation is continuous and the step sizes may not necessarily decrease with time.

### Evidence for strong negative frequency-dependent selection on the inactivation of the sorbitol repressor

**Fig 6:**
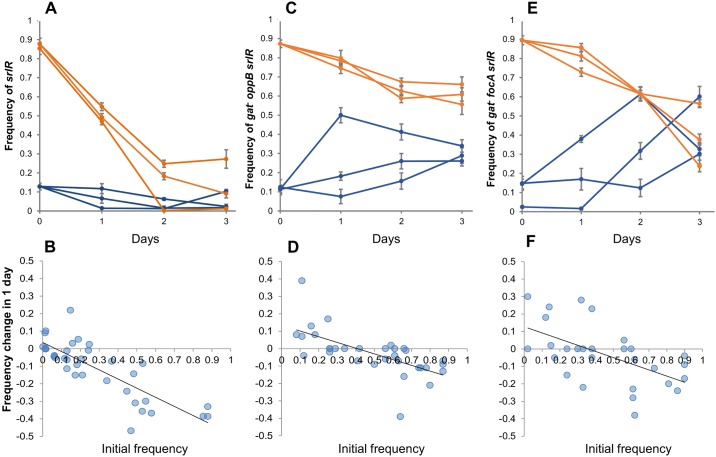
Evidence for balancing selection. *In vivo* competitive fitness assays where the mutant starts at a frequency of ~10% (blue lines) or ~90% (orange lines) in relation to the reference clone. Panel (A) shows the competition between *srlR* and wild type ancestor (DM09); panel (C) shows the competition between 18YFP (*gatC oppB srlR*) and JB19-YFP (*gatC*) and panel (E) shows the competition between 27CFP (*gatC focA srlR*) and JB18-CFP (*gatC*). The temporal dynamics of the frequency of the mutants show negative FDS, maintaining variation at the *srlR* locus, as *srlR* alleles are disadvantageous when at high frequency but are maintained at low frequency (B, D and F, where the change in frequency from day *i* to day *i*+1 as a function of frequency at day *i*, along the temporal dynamics is shown). This form of selection dominates irrespective of the presence of additional mutations, such as those in *focA*, previously determined to be under positive Darwinian selection (Fig 4B).

Multispecies communities of microbes are a likely scenario for the emergence of important ecological interactions. One possible outcome of these interactions is frequency-dependent selection (FDS), a situation where the fitness of a phenotype changes according to its frequency [5]. Negative FDS occurs if a phenotype is deleterious when common but beneficial when rare: this form of selection is capable of maintaining genetic diversity. The results of the competition assays with the *srlR* mutation (Fig 4F) indicate that negative FDS may have played a role in the evolution of the populations. To directly test this hypothesis, we performed new competitive fitness assays starting with different frequencies of the wild type and *srlR* mutant strains, (same genotypes as in Fig 4F). Indeed, *srlR* mutants show a strong disadvantage when competing with the ancestor at high frequency but were maintained at lower frequencies (Fig 6A). When plotting the change in frequency of this mutant as a function of its initial frequency, a significant linear correlation is observed (Fig 6B—Linear regression Adjusted R^2^ = 0.66, P<0.0001; slope = -0.52 (0.06 SE), intercept = 0.04 (0.02 SE), which supports FDS.

Interestingly, the effect of the *srlR* mutation was so strong that frequency dependence was maintained, irrespective of the background in which it occurred. As shown in Fig 6C and 6E, two different genotypes (*gatC oppB srlR* and *gatC focA srlR*), carrying a *srlR* mutation, had a competitive advantage when rare, but a disadvantage when at high frequency (Fig 6D—Linear regression, Adjusted R^2^ = 0.41 P<0.0001; slope = -0.32 (0.07 SE), intercept = 0.13 (0.04 SE) and Fig 6F—Linear regression Adjusted R^2^ = 0.30 P = 0.0015; slope = -0.35 (0.1 SE), intercept = 0.13 (0.05 SE)). This shows that positive Darwinian selection on *focA* (Figs 4B and 5), previously shown to drive this mutation rapidly to fixation, can be highly influenced by negative FDS if the *srlR* mutation co-occurs with this mutation. The strength of selection on *oppB* (Fig 5) is much weaker than that of *focA*, yet when combined with *srlR* it shows a similar dynamics (Fig 6C). This suggests either an epistatic interaction between *srlR* and *oppB* or that *oppB* is also under FDS. Overall, these observations support the hypothesis that the *srlR* mutation *per se* is able to maintain diversity for long periods of time.

### Evidence for periodic selection and clonal interference driving intra-species variation

To monitor adaptation at the genome-wide scale, we analyzed levels of polymorphism across time in two of the evolving populations colonizing the mouse gut. We used both WGS of samples of the populations and typing of selected mutations in clones to unravel the pervasiveness of clonal interference.

**Fig 7:**
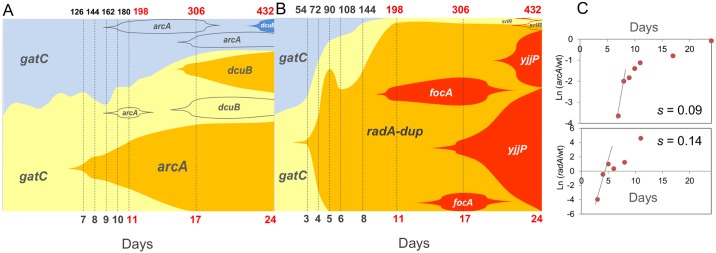
Clonal interference and periodic selection co-occur during adaptation of *E*. *coli* to the mouse gut. Muller plot of the frequencies of newly generated haplotypes during 24 days (corresponding to 432 generations) of evolution inside the mouse gut (see S4 Table for numeric data). Shaded areas are proportional to the frequencies of each haplotype. Yellow and blue shaded areas represent the two sub-populations of bacteria labeled either with *cfp* or *yfp* alleles. Increasingly darker tones of yellow or blue represent the accumulation of target beneficial mutations in clones with a given fluorescence background (either *cfp* or *yfp*). In cases where the fluorescent background is not known, the new mutation (as assayed by whole genome population sequencing) is indicated without change in the shading tone. Evidence for positive Darwinian selection in the gut: clonal interference, where clones carrying different adaptive mutations at the same and in different loci compete for fixation in the gut in population 2.7 (A); periodic selection results in the rapid replacement of a clone, *i*.*e*. a hard selective sweep in which the haplotype *radA*-dup reaches close to 100% frequency in the population 2.10 (B). Dashed lines mark the time points in days (lower axis) or generations (lower axis) where the sampling of clones (in black) or WGS (in red) of the population took place. For population 2.7, between 40 and 50 clones were analyzed at each time point, whereas in population 2.10, between 20 and 60 clones were analyzed each day (please refer to Methods for the further details of this analysis). The effects of two second step mutations (*radA-*dup and *arcA*) were estimated through their frequency increase in the populations where they emerged (C). This quantification was only possible because additional mutations are very unlikely to be segregating at the same time. Only mutations observed more than once in any of the sequenced clones are represented here. Population 2.7 has one additional mutation not represented in the figure and population 2.10 has two other mutations (see S4 Table).

Distinct alleles at each locus were found segregating in the two populations assayed (Fig 7A and 7B and S3 Table): two alleles for *focA*, *yjjP*, *srlR*, three for *dcuB* and four for *arcA*, further supporting the adaptive nature of the identified targets and showing that the evolutionary process is not limited by mutation.

Importantly, population 2.10 is remarkable in that it shows evidence of co-occurrence of all classical forms of natural selection (periodic selection, clonal interference and FDS), within less than a month. First, there is an example of a molecular pattern close to that expected under periodic selection, where a strong haplotype involving an IS insertion in *radA* and a large duplication (*radA*-dup) rapidly sweeps close to fixation (Fig 6B). Second, after day 11 a signature of clonal interference between different alleles of *focA* and *yjjP* is observed. Finally, towards the end of the experiment, clones carrying mutations in *srlR* emerge, which should result in negative FDS (as shown in Fig 6).

The selection coefficient associated with the *radA-dup* mutant can be estimated from its change in frequency while sweeping through the population, but before other mutations can be detected. The estimate for the effect of this mutation is *s*_*rad* dup_ = 0.14 (Fig 7C). This is the highest selection coefficient for all of the mutations of the second steps of adaptation (Fig 5), though it is based on a single observation.

In the same manner, a selective benefit for the *arcA* mutation can also be estimated (Fig 7C). A selection coefficient of 0.09 is estimated between day 7 and 8 when the mutation was first detected and before day 11 when another mutation (*pphB*) was also observed (S4 Table). Even though this is a crude estimate, it is remarkably consistent to that measured in direct competition between a mutant carrying the same IS5 insertion in the *arcA* regulatory region and the ancestor of the second colonization (Fig 6 and S3 Table *s*_*arcA*_ = 0.09±0.02).

## Discussion

The rules governing the adaptive process of microbial populations in natural communities are far from understood. Numerous aspects of host physiology [38] and even behavior are influenced by the microbiota [39], making this a priority environment to appreciate how much microbial evolution occurs within the lifetime of a host. Nevertheless, the amount of evolution taking place in this environment, as well as its contribution to the overall diversity resulting from *de novo* mutation is currently underappreciated. We followed evolution in a natural environment under controlled conditions and studied two consecutive bouts of adaptation. The first bout was caused by the emergence of multiple mutations (both within and among hosts) causing a similar phenotype, loss of function of the operon encoding galactitol metabolism (*gat*-negative phenotype), and an associated selective effect of 0.08 ± 0.01 per generation [8]. The ability to use galactitol is a polymorphic trait in wild strains of *E*. *coli* [40]. The second bout of adaptation targeted at least seven different loci. No strong signals of deceleration in adaptation rate were detected, judging from the strong effect of the second step mutations estimated either from their frequency increase in the evolving populations (Fig 6C, *s*_*rad-dup*_ = 0.14 and *s*_*arcA*_ = 0.09) or from direct *in vivo* competition assays against the ancestral strain of the second colonization, which had a *gat*-negative phenotype (Fig 5). This observation contrasts with the pattern of diminishing returns epistasis previously found in *E*. *coli* adapting to a glucose limiting environment [29] and in *Methylobacterium extorquens* AM1 evolved in batch culture with methanol as the sole carbon source [41]. The extent to which the fitness effects of further mutations that accumulate *in vivo* may become smaller as adaptation proceeds remains to be determined in future work.

By discretizing the adaptive steps we aimed to understand not only the rhythm but also the repeatability of adaptation in the gut environment. WGS of clones isolated from different mice revealed 7 parallel targets comprising: three membrane transporters, one repressor of a metabolic operon, one major regulator involved in the aerobiosis/anaerobiosis transition, one large duplication, and a protein of unknown function. Interestingly all mutations tested, which occurred in regulatory regions, either up-regulated the targeted gene directly, or downstream genes in the regulatory cascade. The fact that *E*. *coli* adapted to the gut environment by up-regulating (directly or indirectly) nutrient transport functions bears an interesting resemblance to the observations of microbes adapting to limiting nutrient conditions in chemostats [42]. For instance, *E*. *coli* adapted to low lactulose chemostats by duplicating the *lac* operon or abolishing its regulation [43] and *Sacharomyces cerevisae* adapted to glucose-limited conditions by amplifying a region including high affinity transporters [44]. Similarities between adaptation in the gut and in chemostats maybe expected to some extent, given that the later were built for continuous culture, where the flow is controlled by a peristaltic pump mimicking digestive transit. Furthermore, chemostats enable conditions to be set to ensure a slow growth rate by manipulating the concentration of the limiting nutrient, perhaps simulating the conditions experienced in the gut by *E*. *coli* [22]. Remarkably, in chemostats with limiting glucose concentrations, a great amount of clonal interference and mutations exhibiting frequency dependence was shown to occur [45,46].

Epistasis is an important factor that can impose strong constraints on the adaptive process of bacterial populations [47,48]. This has been extensively studied *in vitro* [29,30,49,50] but only rarely *in vivo* [51]. In the context of bacterial evolution in the gut we evaluated whether epistasis contributed to the order of the adaptive steps that characterized *E*. *coli* evolution during colonization of the mouse intestine. Our setup provides an ideal situation to address this question, since the order of adaptive events was absolutely conserved with a single universal first adaptive phenotype. One possible explanation for the order of events could result from the second mutations being weaker or even deleterious in the ancestral genotype. In fact we estimated that at least one third of the second step mutations were as strong as those responsible for the first step, showing that strong effect mutations were still available for adaptation. However, many showed a smaller average effect (mean *s*_(*oppB*, *yjjP*,, *dcuB*, *focA*_) = 0.03). Therefore, it is possible that the large effect size of the first-step may have contributed for the order observed. A much more likely explanation can, however, be provided by differences in mutation rate. Indeed, we found that the *gat* operon is inactivated at a much higher rate than a random locus (10^−5^ versus 3.6x10^-8^). This fact very likely contributed to the 100% parallelism observed at the phenotypic level, inactivation of the galactitol metabolism, despite the much reduced parallelism at the genetic level [8]. Besides the *gat*, shown here, other metabolic operons have been previously found to be mutational hotspots. One example is the *ribose* operon which was the most common target in one experiment of *E*. *coli* adaptation to glucose minimal medium [52]. Inactivation of this operon occurred at a high rate (~5 x 10^−5^ per genome, per duplication), similar to that of *gat*. However it conferred a 1–2% benefit to *E*. *coli* growing on glucose, considerable smaller than the 8% benefit conferred by *gat* inactivation in *E*. *coli* colonizing the mouse gut.

Overall we present some of the first quantitative estimates of the fitness effects of beneficial mutations occurring in bacteria colonizing a natural ecosystem, where both ecological and evolutionary processes occur at fast time scales. The data obtained establish that strong effect beneficial mutations exhibiting all classical forms of natural selection shape the genetic diversity of a commensal species inhabiting the mouse gut microbiota. They show that, even in mammalian hosts with identical genetic backgrounds and diets, reproducible adaptations emerge albeit with a significant level of host individuality.

## Materials and Methods

### Ethics Statement

All experiments involving animals were approved by the Institutional Ethics Committee at the Instituto Gulbenkian de Ciencia (project nr. A009/2010 with approval date 2010/10/15), following the Portuguese legislation (PORT 1005/92), which complies with the European Directive 86/609/EEC of the European Council.

### Bacterial Strains and culture conditions

All strains used were derived from MG1655, a K12 commensal strain of *Escherichia coli* [53]. Strains JB19-YFP and JB18-CFP (MG1655, *galK*::*YFP/CFP* cm^R^, str^R^ *(rpsL150)*, *ΔlacIZYA*, Ins (1bp) *gatC*) were used for the evolution experiment here reported. These strains differ from the ancestral MG1655 fluorescent strains DM08-YFP and DM09-CFP used in a previous evolution experiment (MG1655, *galK*::*YFP/CFP* amp^R^, str^R^ *(rpsL150)*, *ΔlacIZYA)*[9]) by a mutation in the *gatC* gene (1bp insertion in the coding region). To construct these strains the ampicillin resistance cassette in the ancestral strains DM08-YFP and DM09-CFP was replaced with a chloramphenicol resistant cassette using the Datsenko and Wanner method. The yellow (*yfp*) and cyan (*cfp*) fluorescent genes linked to cm^R^ were then transferred by P1 transduction to a derivative of clone 12YFP [8], an evolved clone of DM08-YFP, isolated after 24 days of adaptation in the gut of WT mice, that carried an insertion of 1bp in *gatC* and a large duplication. During the genetic manipulations the large duplication was lost, confirmed by whole genome sequencing, leaving this clone with a single mutation, 1bp insertion in *gatC*.

To measure the effect of the identified parallel mutations in gene expression we tested the following clones isolated from the evolution experiment reported here (see S1 Table): 18YFP (*focA srlR*), 22YFP (*dcuB*), 25YFP (*yjjP/yjjQ radA insX-insA*), 29CFP (*arcA*) and the ancestral strains DM08-YFP and DM09-CFP. Five biological replicates from each clone were performed for both aerobic and anaerobic conditions.

To directly measure the effects of the mutations involved in the 2^nd^ step of adaptation (in the *gat*-positive background) through *in vivo* fitness assays we used several single mutant clones isolated from a previous evolution experiment [8] (5YFP (*srlR*) and 6YFP (*dcuB*)) or the experiment here reported (17YFP (*arcA*), and 2 clones isolated from population 2.14 screened by PCR for mutation at *focA* and *yjjP* locus, respectively). The *oppB* single mutant was obtained by transducing the knockout of this gene (*oppB*::*Kan*) from the KEIO collection [54] to the ancestral YFP background. These clones, initially carrying an additional mutation in one of *gat* operon genes (*gatA* or *gatC*), were made single mutants by P1 transduction from JW2074 (*gatR*::Kan), a *gatR* knockout mutant from the KEIO collection. *gatR* is already interrupted by a transposable element in the ancestral MG1655 strain and therefore transduction with P1 from a mutant strain *gatR*, results in the effective replacement of the neighbor genes of the *gat* operon to their wild type status, while maintaining a knockout mutation in *gatR*. As reference strains, we used derivatives of the ancestral DM08-YFP and DM09-CFP strains in which the *gatR* gene was replaced with *gatR*::Kan.

All mutants used to test the effect of the 2^nd^ step mutations in the *gat*-negative background were derived from the collection of mutants constructed to test the effect of the same mutations in the *gat*-positive background. These mutants, except for *srlR*, were made *gat*-negative by P1 transduction of the *gatC* allele present in the ancestor of the 2^nd^ colonization. Selection of *gat*-negative mutants was achieved by incorporating 10mM of D-Arabitol in the selection plates. All clones were confirmed to have the intended gatC allele by target PCR and restriction analysis as described in [8]. The *srlR* mutant was obtained by transduction of the *gatC*::Kan from the KEIO collection and was competed against a reference strain with the same *gatC*::Kan.

To test for FDS we competed the single *srlR* mutant previously mentioned (derived from clone 5YFP but with a wild type *gat* operon), against the ancestral strain DM09 (MG1655-CFP). We also competed the clones 18YFP (*gatC focA srlR*) and 27CFP (*gatC oppB srlR*) against the reference strains JB18-CFP (*gatC*) and JB19-YFP (*gatC*), respectively.

In order to test if clones evolved *in vivo* had a different growth ability we performed *in vitro* competitions against the ancestral strains DM08-YFP and DM09-CFP of clones isolated from 14 independent populations from a previous evolution experiment (sequenced clones 1 to 14 from populations 1.1 to 1.14) and this evolution experiment (sequenced clones 16 to 30, from populations 2.1 to 2.15, S1 Table).

*In vitro* competitions to measure the effect of the *gat* operon and *nfsA* gene inactivation were performed using the clones 4YFP [8] and a clone obtained by P1 transduction of *nfsA*::Kan (from the KEIO collection), DM08-YFP and DM09-CFP. Mutant clones were constructed in the two fluorescent backgrounds and competed against the ancestors DM08-YFP or DM09-CFP, depending on the fluorescent background.

To distinguish between *gat*-negative and *gat*-positive bacteria we used the differential medium MacConkey agar supplemented with galactitol 1% and streptomycin (100μg/ml). Plates were incubated at 30°C. The frequency of galactitol mutants was estimated by counting the number of white (auxotrophic for galactitol) and red colonies.

To perform the fluctuation test for the *gat*-negative phenotype we used the selective M9 Minimal Medium (MM) agar, supplemented with D-arabitol (10mM) and glycerol (0.4%) or Luria Broth (LB) agar supplemented with furazolidone (1.25 μg/ml).

For the *in vitro* competition assays we used MM supplemented with 3mM of MgSO_4_ and either sorbitol, ribose, mannose, gluconate or glucuronate at a concentration of 0.02%. Additionally a mixture with the different carbon sources (composed of 0.1% from each of the five carbon sources) was also used.

### Evolution experiment

In order to study *E*. *coli*´s adaptation to the gut we used the classical streptomycin-treated mouse colonization model [55] and performed the evolution experiment using the same conditions as before [8]. Briefly, 6- to 8-week old C57BL/6 male mice raised in specific pathogen free (SPF) conditions were given autoclaved drinking water containing streptomycin (5g/L) for one day. After 4 hours of starvation for water and food, the animals were gavaged with 100μl of a suspension of 10^8^ colony forming units (CFUs) of a mixture of MG1655-YFP-*gatC* and MG1655-YFP-*gatC* bacteria (ratio 1:1) grown at 37°C in brain heart infusion medium to OD_600_ of 2. After the gavage, all the animals were housed separately and both the water with streptomycin and the food were returned to them. Mice fecal samples were collected for 24 days and diluted in PBS, from which a sample was stored in 15% glycerol at -80°C and the remaining was plated in Luria Broth agar supplemented with streptomycin (LB plates). Plates were incubated overnight at 37°C and then with the help of a fluorescent stereoscope (SteREO Lumar, Carl Ziess) the fluorescent colonies were counted to assess the frequencies of CFP- and YFP-labelled bacteria. These fluorescent proteins are used as neutral markers with which we can follow the appearance of beneficial mutations, since these markers hitchhike with the beneficial mutations that spread in the populations [9].

### *In vivo* competitive assays

To test the *in vivo* advantage of 12 clones carrying the 2^nd^ step mutations in (n = 4 per clone) (Figs 4 and 5) and 3 clones carrying the *srlR* mutation (n = 3 per clone) we performed competitive assays against the respective ancestor labelled with the opposite fluorescent marker. *In vivo* competitions were performed at a ratio of 1 to 1 for all clones except the ones where we tested for FDS. To test for FDS we performed ratios of 1:9 and 9:1, following the same procedure described above for the evolution experiment. Mice fecal pellets were collected daily, diluted in PBS and frozen in 15% glycerol at -80°C. Total numbers and relative proportions of YFP- and CFP-labeled *E*. *coli* were subsequently determined by plating appropriate dilutions in either LB agar supplemented with streptomycin (100 μg/ml) or MacConkey supplemented with streptomycin (100 μg/ml) and galactitol 1%. After overnight incubation at 30°C, the colonies were screened for the *gat* phenotype, based on their white or red color. In addition, CFP- and YFP-labelled bacteria were counted with a fluorescent stereoscope (SteREO Lumar, Carl Ziess).

The selection coefficient (fitness gain) of the clones *in vivo* (presented in Fig 1) was estimated as: $s_{b}=\text{ln}\left(\frac{Rf_{ev/anc}}{Ri_{ev/anc}}\right)/t$, where *s*_*b*_ is the selective advantage of the evolved clone, *Rf*_*ev/anc*_ and *Ri*_*ev/anc*_ are the ratios of evolved to ancestral bacteria in the end (*f*) or in the beginning (*i*) of the competition and *t* is the number of generation per day. We assumed *t* = 18, in accordance with the 80 minute generation time estimated in previous studies on *E*.*coli* colonization of streptomycin-treated mouse [56,57].

### *In vitro* tests for changes in growth ability of evolved clones

To test whether *E*. *coli* clones evolved *in vivo* had a different nutritional profile when growing *in vitro*, we performed competitions between each of the sequenced clones (obtained both from [8] and from the present work) and the ancestor of the first colonization of the opposite fluorescence. Competitions were performed in triplicate, in MM supplemented with different carbon sources and in two different oxygen conditions. All competitions were conducted in 96-well plates incubated at 37°C (Thermoshaker PHMP-4, Grant) under aerobiosis or in an anaerobic chamber (anaerobiosis). The competitor and reference strains were initially acclimated to the growth media for two overnights in MM supplemented with glycerol (0.02%) and then 10^5^−10^6^ cells of both strains inoculated in MM containing 0.02% of either sorbitol, ribose, mannose, gluconate or glucuronate or a mixture of all five carbon sources at individual concentrations of 0.01%. The strains were allowed to compete for 24 hours and the initial and final ratios of both strains were determined by flow cytometry, using a BD LSRFortessa (BD Biosciences) cytometer. The relative fitness of the evolved clones (S2 Table) was estimated as previously described (see “*In vivo* competitive assays” above).

Competitions in anaerobic conditions were performed for each of the evolved clones following the protocol above described but with the following alterations: after an initial aerobic growth overnight in MM with glycerol (0.02%), the cultures were diluted 10-fold, inoculated in MM with glycerol and acclimated overnight by incubation in an anaerobic chamber (5% H_2_, 15% CO_2_, 80% N_2_) (Plas Labs, Lansing, MI, USA), at 37°C. After acclimatization, the competitor and reference strain were inoculated in MM supplemented with individual or a mixture of carbon sources and allowed to compete for 48 hours. To determine the initial and final ratios of the competing strains, serial dilutions of the mixtures were plated in LB supplemented with streptomycin (100μg/ml) and the resulting CFUs counted in a stereoscope (SteREO Lumar, Carl Zeiss).

### Whole genome re-sequencing and mutation prediction

*Clone analysis*: After 24 days of colonization one clone from each independently evolving populations (2.1 to 2.15) was isolated and grown in 10 mL of LB at 37°C with agitation for DNA extraction (following a previously described protocol [58]). The DNA library construction and sequencing was carried out by BGI. Each sample was pair-end sequenced on an Illumina HiSeq 2000. Standard procedures produced data sets of Illumina paired-end 90 bp read pairs with insert size (including read length) of 470 bp. Mutations were identified using the BRESEQ pipeline [59]. To detect potential duplication events we used ssaha2 [60] with the paired end information. This is a stringent analysis that maps reads only to their unique match (with less than 3 mismatches) on the reference genome. Sequence coverage along the genome was assessed with a 250 bp window and corrected for GC% composition by normalizing by the mean coverage of regions with the same GC%. We then looked for regions with high differences (>1.4) in coverage. Large deletions were identified based on the absence of coverage. For additional verification of mutations predicted by BRESEQ, we also used the software IGV (version 2.1) [61]. Data presented in S1 Table.

*Population analysis*: DNA isolation was obtained in the same way as described above for the clone analysis except that now it derived from a mixture of >1000 clones per population grown in LB agar. Two populations, from the evolution experiment, were sequenced: 2.7 and 2.10. Those were sequenced for three time points during the adaptive period (generation 198 (day11), generation 306 (day17) and generation 432 (day24)). The DNA library construction and sequencing was carried out by the IGC genomics facility. Each sample was pair-end sequenced on an Illumina MiSeq Benchtop Sequencer. Standard procedures produced data sets of Illumina paired-end 250 bp read pairs. The mean coverage per sample was between ~90x and ~150x for population 2.7 and between ~100x and ~120x for population 2.10. Mutations were identified using the BRESEQ pipeline (version 0.26) with the polymorphism option on. The default settings were used except for: a) requirement of a minimum coverage of 3 reads on each strand per polymorphism; b) eliminating polymorphism predictions occurring in homopolymers of length greater than 3; c) polymorphism predictions with significant (*P* = 0.05) strand or base quality score bias were discarded. Data presented in S3 Table.

### Analysis of gene expression changes caused by IS insertions

To determine the effects of the IS insertions identified during the 2^nd^ steps of adaptation we measured the expression of *focA*, *dcuB*, *arcA*, *yjjY* and *yjjP* by RT-qPCR in two environments with different levels of oxygen. Five biological replicates and three technical replicates per clone were performed.

*Aerobic Conditions*: The clones were initially grown for 24h at 37°C with aeration in MM with glycerol (0.02%). The cultures were diluted 10-fold and 100 μl of the dilution were inoculated in in 10ml of M9 minimal medium (MM) supplemented with a mixture of the following carbon sources: sorbitol, ribose, mannose, gluconate and glucoronate, at individual concentration of 0.01%. The cultures were grown at 37°C, with aeration, until an OD_600_ of 0.5. Five milliliters of the bacterial culture were then harvested by centrifugation at 4°C for 5 minutes at the maximum speed. The resulting pellet was ressuspended in lysozyme solution (5 mg lysozyme /ml DEPC treated water, Sigma protocol) and incubated at 37°C for 30 minutes, promoting disruption of the bacterial cell wall and allowing for RNA extraction (see below).

*Anaerobic conditions*: The protocol used was the same as in the aerobic conditions with the following alterations: the second overnight growth was performed at 37°C in an anaerobic chamber with the atmosphere of 5% H_2_, 15% CO_2_, 80% N_2_ (Plas Labs, Lansing, MI, USA), and at approximately OD_600_ of 0.2 the cultures were placed in dry ice to prevent their growth and the cells were harvested by centrifugation from 10 ml of bacterial culture.

*RNA extraction*, *DNAse treatment*, *RT-PCR and qPCR*: The RNA extraction was performed with the Qiagen RNeasy Mini Kit. RNA concentration and quality were evaluated with Nanodrop 2000. DNase treatment was performed with the RQ1 DNase (Promega), 0.5μl of DNase and 1μl buffer were added to 1μg of RNA and incubated for 30 minutes at 37°C. After this, 1μl stop solution was added and then incubated for 15 minutes at 65°C to inactivate the DNase. The resulting RNA was used for the reverse transcription which consisted in mixing with 1μg of RNA, with 0.5μl random primers and DEPC-water (final volume of 15μl) and then incubated at 70°C for 5min. Afterwards the M-MLV Reverse Transcriptase Protocol (Promega) were performed, to the first mix was added 5 μl of RT buffer, 0.5μl RT enzyme and 2μl dNTP mix, and then incubated 10 min at 25°C, 50min at 50°C and 10 min at 70°C.

We used a relative quantification method of analysis with normalization against a reference gene. qPCR was executed in BioRad CFX 384 with itaq universal sybr green supermix (BioRad). cDNA was diluted 100-fold before used in the qPCR. The qPCR reaction conditions were as follows: one cycle of 2 min at 50°C and then 39 cycles of 10 min at 95°C, 30 sec at 95°C, 1 min at 57°C and finally 30 s at 72°C. Primers used are listed in S5 Table. Melting curve analysis was performed to verify product homogeneity. All reactions included three replicates for each sample. Data were normalized by the Pfaffl method [19] using the *hfq* housekeeping gene of *E*. *coli* as a reference.

### Fluctuation test for the *gat*-negative phenotype

To test for the possibility of a difference in the mutation rate of the galactitol operon, we determined the frequency of spontaneous *gat*-negative phenotype mutants when plated on D-arabitol. D-arabitol is known to be toxic for bacteria that are able to metabolize galactitol (*gat*-positive phenotype) [62] and so the growth of *gat*-positive bacteria is much slower, allowing to differentiate between *gat*-positive and *gat*-negative clones. The ancestral strains DM08-YFP and DM09-CFP were grown overnight in 10 ml of LB at 37°C with aeration. After growth, the total number of cells in the cultures was measured using BD LSR Fortessa (BD Biosciences) and approximately 1000 cells were used to inoculate 1 ml of LB (10 replicates of each strain) and incubated overnight. Aliquots of each replicate tube were plated in LB agar and MM agar supplemented with D-arabitol (10 mM) and glycerol (0.4%) and incubated overnight at 37°C. The number of spontaneous *gat*-negative mutants and total number of cells grown on LB were used to estimate the mutation rate using the maximum likelihood approach as implemented in FALCOR [25].

Similarly, a fluctuation assay for measuring the spontaneous rate of emergence of furazolidone resistant mutants was used as proxis for the spontaneous rate of random gene inactivation. We then used this number to compare with the rate for *gat*-negative phenotype. The experiment was performed in the same conditions as described above except that the cultures were plated in LB supplemented with furazolidone (1.25μg/ml).

### Identification of adaptive mutations and estimate of haplotype frequencies in selected populations of the evolution experiment

In order to estimate the haplotype frequencies depicted in Fig 6A and 6B two complementary strategies were employed. In addition to the WGS of the populations, targeted PCR of the identified parallel mutations was performed. For the targeted PCR, 20 to 80 clones from different time points were screened (from populations 2.7 and 2.10) using the same primers and PCR conditions as in [8]. Because all target mutations correspond to IS insertions an increase in the PCR band is indicative of the presence of an IS. Frequencies are depicted in S3 Table.

### Statistical analysis

To determine significant differences in gene expression between mutant and ancestral strain, the unpaired t test was used, with a significance defined as *P* value of <0.05.

Differences in the selective advantage of clones competed *in vitro* against the ancestral were evaluated with paired one-tailed distribution t test, with a significance defined as *P* value of <0.05. All statistical analysis were conducted with the statistical software R [63].

### Accession Numbers

Genome sequencing data have been deposited in the NCBI Read Archive, http://www.ncbi.nlm.nih.gov/sra (accession no. SRP063701).

## Supporting Information

S1 Fig Position of the adaptive IS insertions. Exact position (except for the small sequence duplicated upon IS insertion, shaded in green) of the IS insertions in the regulatory regions of *dcuB*, *focA*, *arcA* and *yjjP*. The genomic coordinates of the sequences represented are indicated between brackets. The start codon of each gene is shaded in yellow and marked with the letter M (methionine). Arrows represent positions of transcription start site. Boxed sequences represent binding sites for transcriptional activators (in green) or repressors (in red). D, N, F, C, A, I, H correspond to the following DNA-binding transcriptional dual regulators: DcuR-Phosphorylated, NarL Phosphorylated, FNR, CRP-cAMP, ArcA-phosphorilated, IHF and H-NS. The sequence annotation is according to EcoCyc [64].
(TIF)

S2 Fig Dynamics of the 2^nd^ step mutations competing in the two backgrounds (*gat*-positive and *gat*-negative) against the respective ancestors. Shown are the frequencies of the 2^nd^ step mutations, labelled with the *yfp* allele, along 3 days of competition. (A) Competitions in the *gat*-positive background. (B) Competitions in the *gat*-negative background. The natural logarithm of the ratio of each mutant to the ancestor over the first 3 days of competition was used to estimate the selection coefficients depicted in S3 Table and Fig 5. Four independent competitions were performed to test each mutation. Error bars represent 2SE.
(TIF)

S1 Table Identity of the mutations detected in the sequenced clones. Mutations were identified by comparison with the ancestor. The ancestor differs from (NCBI Reference Sequence: NC_000913.2) in the positions reported in (1) for the 0YFP clone plus 1pb insertion (+C) in the coordinate 2172079 (*gatC*, coding (222/1356 nt)). Mutations in intergenic regions, as for example *yjjY/yjtD*, list the two flanking genes. Numbers in the annotation row represent nucleotides relative to each of the neighboring genes, where + indicates the distance downstream of the stop codon of a gene and—indicates the distance upstream of the gene, that is relative to the start codon. Genes inside brackets means that the mutation is localized within the gene. Single nucleotide substitutions (SNP) are represented by an arrow between the ancestral and the evolved nucleotide. Whenever a SNP gives rise to a non-synonymous mutation the amino acid replacement is also indicated. One asterisk means that the corresponding SNP originated a STOP codon. The symbol Δ means a deletion event and a + symbol represents an insertion of the nucleotide that follows the symbol. IS denotes the abbreviation of insertion sequence element at the indicated position.
(XLSX)

S2 Table Phenotypic characterization of gut adapted clones. *In vitro* competitive fitness assays of evolved clones against the ancestral shows increased fitness when growing under presence or absence of oxygen in several carbon sources present in the mouse intestine. Numbers represent the selection coefficient (*s*) of each clone in relation to the reference strain (see Material and Methods for details on how we performed the competitions and estimate *s*). Yellow, green and red shading means that *s* is not significantly different, is higher or lower than the ancestral clone, respectively. The column “Genotype” depicts the genes affected by mutation in each clone. Clones are ordered by genotype similarity.
(XLSX)

S3 Table Selection coefficients of the 2^nd^ step mutations. Rows named “*s* (*gat*-pos)” and “*s* (*gat*-neg)” show the selection coefficients of the 2^nd^ step mutations (listed in the first row) measured in direct competition (*in vivo*) with the respective ancestors (see also the S2 Fig and Fig 5). The error estimates associated with each measurement represents 2SE. Shaded in red are the estimates in the *gat*-positive background and in blue in the *gat*-negative. The asterisks denote the significance of the pairwise comparisons of the effects of mutations in the each of the backgrounds (ANOVA with Tuckey correction for multiple comparisons. * *P* ≤ 0.05, ** *P* ≤ 0.01 and *** *P* ≤ 0.001). The diagonal shows the comparison of the effect of the same mutation in the two backgrounds (Mann-Whitney-Wilcoxon). The green rectangle highlights a mutation with positive epistasis with the background whereas the red triangle highlights negative epistasis. N.A. means not applicable. N.S. stands for non-significant.
(XLSX)

S4 Table Genetic polymorphism in two evolved populations. Mutations were detected by whole genome sequencing of samples of each population at different time points (shown in red) or by target sequencing (shown in black. The full data is graphically represented in Fig 5.
(XLSX)

S5 Table Primers used for the RT-qPCR experiment. The first row indicates the gene targeted by the qPCR primers. The resulting measurements, relative expression levels of the target genes, are depicted in Fig 2.
(XLSX)
