# Supplementary figures and images for: A Mutational Hotspot and Strong Selection Contribute to the Order of Mutations Selected for during Escherichia coli Adaptation to the Gut
Source: PLoS Genet. 2016 Nov 3;12(11):e1006420. doi: 10.1371/journal.pgen.1006420 (PMC5094792; doi:10.1371/journal.pgen.1006420)

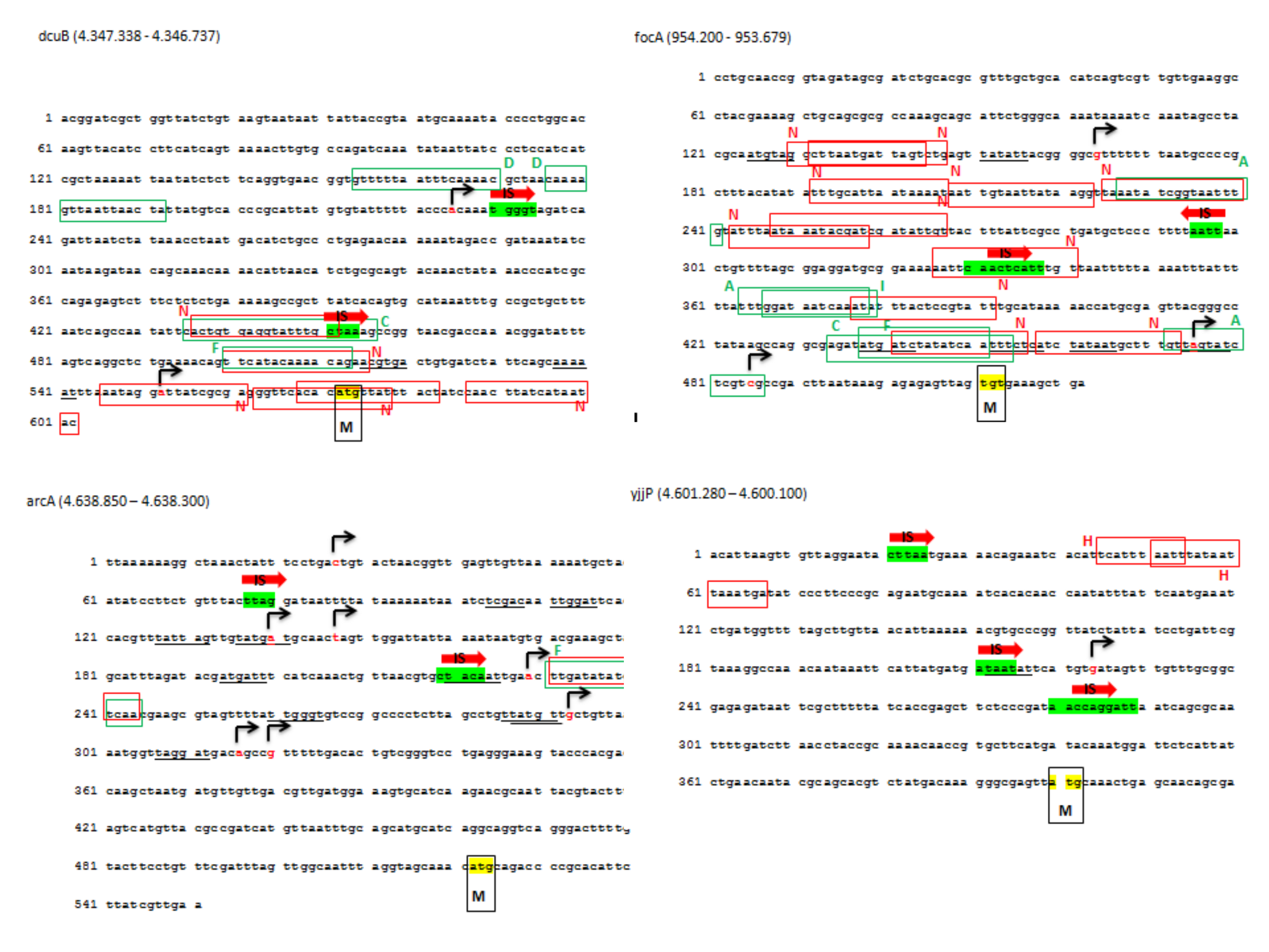

Supplement: S1 Fig — Exact position (except for the small sequence duplicated upon IS insertion, shaded in green) of the IS insertions in the regulatory regions of dcuB, focA, arcA and yjjP. The genomic coordinates of the sequences represented are indicated between brackets. The start codon of each gene is shaded in yellow and marked with the letter M (methionine). Arrows represent positions of transcription start site. Boxed sequences represent binding sites for transcriptional activators (in green) or repressors (in red). D, N, F, C, A, I, H correspond to the following DNA-binding transcriptional dual regulators: DcuR-Phosphorylated, NarL Phosphorylated, FNR, CRP-cAMP, ArcA-phosphorilated, IHF and H-NS. The sequence annotation is according to EcoCyc [64]. (TIF) [file pgen.1006420.s001.tif]

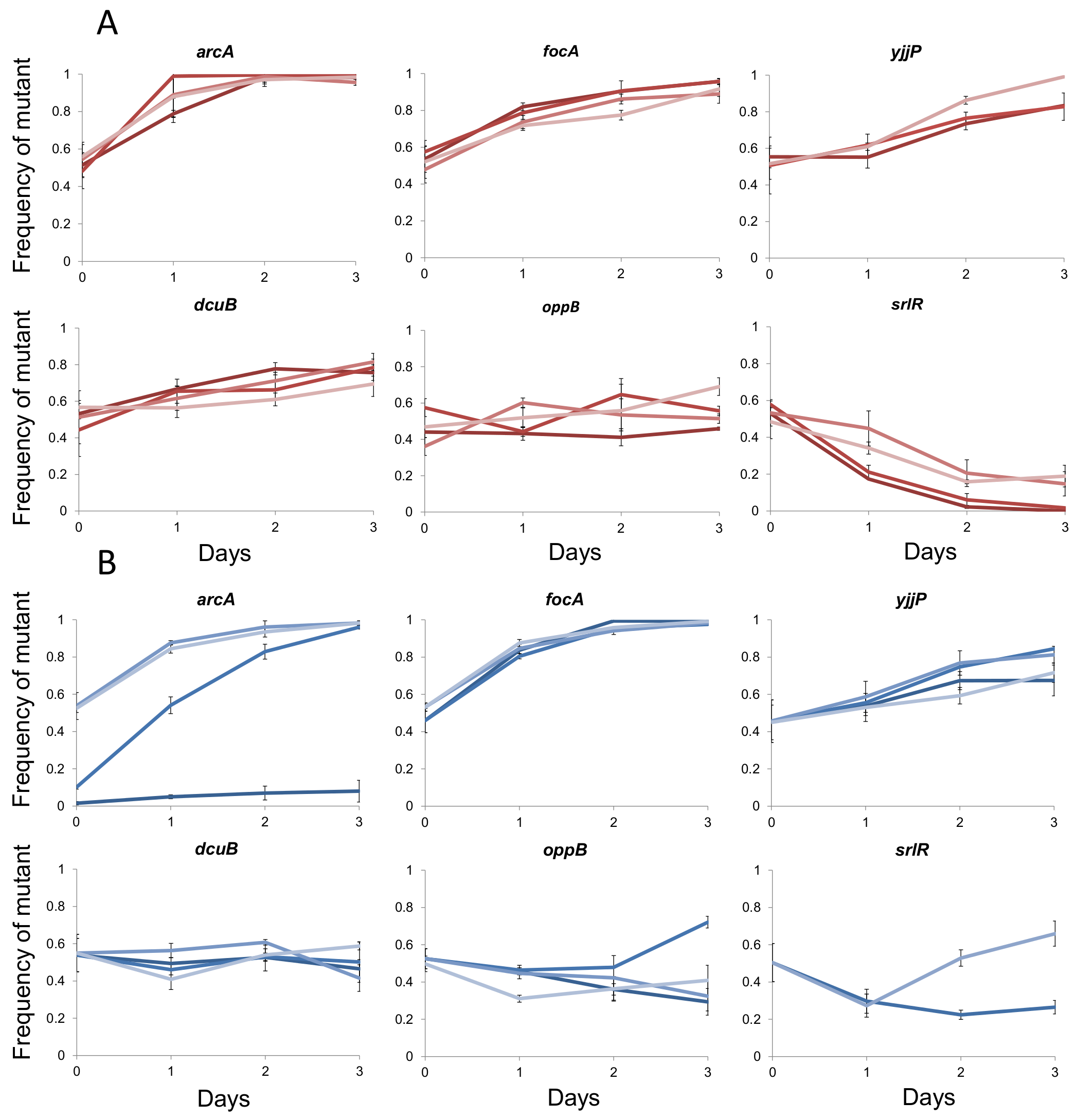

Supplement: S2 Fig — Shown are the frequencies of the 2nd step mutations, labelled with the yfp allele, along 3 days of competition. (A) Competitions in the gat-positive background. (B) Competitions in the gat-negative background. The natural logarithm of the ratio of each mutant to the ancestor over the first 3 days of competition was used to estimate the selection coefficients depicted in S3 Table and Fig 5. Four independent competitions were performed to test each mutation. Error bars represent 2SE. (TIF) [file pgen.1006420.s002.tif]
